# Deciphering Radiotherapy Resistance: A Proteomic Perspective

**DOI:** 10.3390/proteomes13020025

**Published:** 2025-06-16

**Authors:** Davide Perico, Pierluigi Mauri

**Affiliations:** 1 Institute of Biomedical Technologies-National Research Council ITB-CNR, Via Fratelli Cervi 93, 20054 Milan, Italy; davide.perico@cnr.it; 2 Institute of Endotypes in Oncology, Metabolism and Immunology “G.Salvatore”-National Research Council IEOMI-CNR, Via Sergio Pansini 5, 80131 Napoli, Italy

**Keywords:** proteomics, radioresistance, biomarkers

## Abstract

Radiotherapy resistance represents a critical aspect of cancer treatment, and molecular characterization is needed to explore the pathways and mechanisms involved. DNA repair, hypoxia, metabolic reprogramming, apoptosis, tumor microenvironment modulation, and activation of cancer stem cells are the primary mechanisms that regulate radioresistance, and understanding their complex interactions is essential for planning the correct therapeutic strategy. Proteomics has emerged as a key approach in precision medicine to study tumor heterogeneity and treatment response in cancer patients. The integration of mass spectrometry-based techniques with bioinformatics has enabled high-throughput, quantitative analyses to identify biomarkers, pathways, and new potential therapeutic targets. This review highlights recent advances in proteomic technologies and their application in identifying biomarkers predictive of radiosensitivity and radioresistance in different tumors, including head and neck, breast, lung, and prostate cancers. Sample variability, data interpretation, and the translation of findings into clinical practice remain challenging elements of proteomics. However, technological advancements support its application in a wide range of topics, allowing a comprehensive approach to radiobiology, which helps overcome radiation resistance. Ultimately, incorporating proteomics into the radiotherapy workflow offers significant potential for enhancing treatment efficacy, minimizing toxicity, and guiding precision oncology strategies.

## 1. Introduction

Cancer represents one of the biggest threats to public health, and despite advancements in its comprehension and treatment, it remains one of the main causes of death, with growing prevalence [1]. Radiotherapy or radiation therapy (RT) is a key strategy against tumors, often in combination with surgery, chemotherapy, and immunotherapy, contributing to the care and treatment of approximately half of cancer patients [2]. RT is frequently used in combination with surgery through an adjuvant approach as additional treatment post-surgery to remove residual cells and reduce the risk of recurrence. Otherwise, preoperative neoadjuvant radiotherapy is used to reduce tumor size and enhance the chances of successful surgery.

RT uses high-energy radiation to kill or damage cancer cells, preventing their growth and division, and its primary goal is to target cancer cells while minimizing damage to surrounding healthy tissues. Other benefits include non-invasiveness, symptom relief, and immunogenic potential through the abscopal effect [3]. On the other hand, primary side effects include fatigue, skin irritation, and localized pain, in addition to dose-limit tolerance and the long-term risk of developing secondary cancers. Additionally, metastatic and radioresistant cancers [4] may not respond effectively to radiotherapy and can lead to relapse and poor survival.

The mechanism of action of radiotherapy involves direct damage to the DNA of cancer cells, typically through single- and double-strand breaks [5], and indirect damage by the production of reactive oxygen species (ROS) and free radicals that cause oxidative stress [6]. The consequences include the activation of several pathways, such as DNA-damage response (DDR), cell cycle arrest, and apoptosis, resulting in cancer cell death.

**Figure 1:**
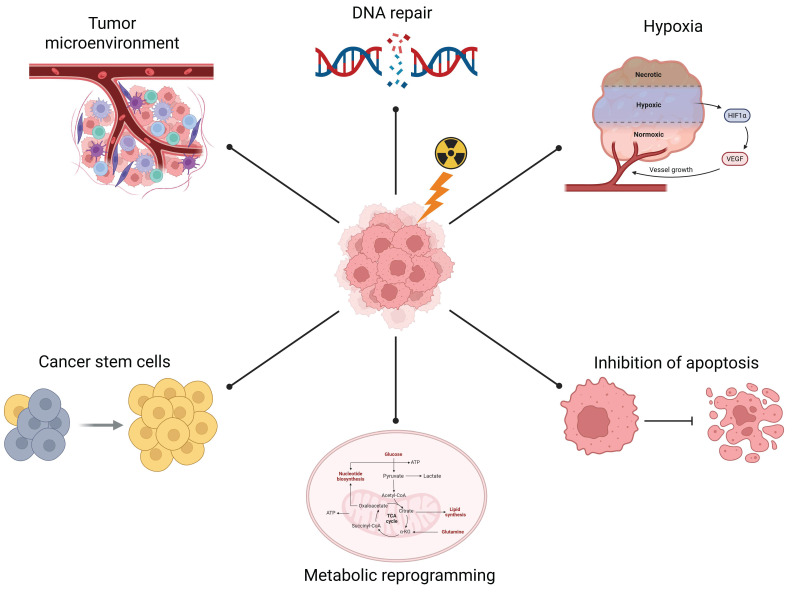
Primary mechanisms of radiotherapy resistance. Created in BioRender. Perico, D. (2025) https://BioRender.com/82u1fab, accessed on 14 April 2025.

Radiation resistance, primary or acquired, allows cancer cells to escape this fate, resulting in tumor recurrence and metastasis [7] and so becomes a challenge to the long-term survival of patients who have undergone RT. Several mechanisms (Figure 1) by which a tumor is or becomes resistant to radiotherapy were proposed [8], and include several factors such as enhanced DNA repair, hypoxia, angiogenesis, and the action of the tumor microenvironment and cancer stem cells (CSCs). Hence, overcoming radiation resistance is essential to planning the best treatment strategy and reducing side effects to reach positive therapeutic outcomes.

For these reasons, a molecular and precision medicine approach is fundamental for understanding radiation resistance mechanisms and identifying related biomarkers to stratify patients, therefore developing appropriate treatment strategies tailored to the subjects and their response to radiotherapy [9,10]. In this field, omics technologies, used in the context of a systems biology approach [11], can provide key information regarding the identification and quantification of molecular components of cells, tissues, and organs, enabling the description of the molecular interactions and unraveling the systems’ functionalities. High-throughput omics technologies such as genomics, transcriptomics, proteomics, and metabolomics [12] are becoming increasingly important in clinical practice for the study of multifactorial and complex diseases like cancer [13], and their application combined with physiological, biochemical, and clinical data and environmental factors can be used by systems medicine to create models useful for biomarker identification and improving stratification, prediction, diagnosis, and therapy.

Proteomics, one of the various omics technologies, focuses on studying the complete set of proteins present in a biological system to gain insights into cellular functions and molecular mechanisms [14,15]. Proteins are the primary drivers of biological processes, and the proteome is more complex than the genome due to the multitude of proteoforms derived from a single gene and the post-translational modifications they undergo. This complexity is further influenced by the specific timing, cellular compartments, and physiological or pathological conditions under which different proteoforms are produced. Hence, proteomic analysis enables the identification and quantification of proteins that contribute to specific phenotypes at precise moments, often linked to diseases or therapeutic interventions. Clinical proteomics plays a crucial role in discovering potential diagnostic and prognostic biomarkers, identifying novel therapeutic targets, and stratifying patients based on molecular profiles, helping to reach preventive and personalized medicine [16,17]. Over the last few decades, liquid chromatography coupled with tandem mass spectrometry (LC-MS/MS) has emerged as the preferred technology in proteomics [18]. This method combines the robust separation capabilities of high-performance liquid chromatography for complex mixtures with the high sensitivity and resolution of mass spectrometry for protein identification.

In clinical research, proteomics can explore the molecular mechanisms of pathologies and the cellular changes induced by diseases, treatments, or environmental factors, comparing different protein profiles to uncover qualitative and quantitative differences. Based on these premises, proteomics can help radiotherapy applications by investigating proteins and pathways related to radiation resistance [19,20]. This approach enables the identification of biomarkers that can predict RT sensitivity and discriminate between responder and non-responder patients, allowing the best treatment plan and a positive outcome.

This review summarizes the applications of MS-based proteomic techniques in radiotherapy resistance, exploring the pathways and biomarkers involved that can improve therapy towards precision medicine.

## 2. Mechanisms Involved in Radiotherapy Resistance

Cancer cells hamper radiotherapy effects through enhanced DNA repair, antioxidant defenses, apoptosis evasion, metabolic shifts, and microenvironmental remodeling. Proteomic applications focused on total proteome, post-translational modifications, or interactomes can capture radiation-induced alterations in repair proteins, signaling kinases, and novel regulators. By comparing resistant versus sensitive profiles, proteomics helps in the discovery and validation of biomarkers and actionable targets, revealing the molecular changes that drive radioresistance and guiding combination therapies to restore treatment sensitivity.

### 2.1. DNA Damage and Repair

Radiation kills cells by inducing DNA damage through single-strand breaks (SSBs), double-strand breaks (DSBs), and ROS-mediated base damage. These lesions trigger signaling cascades of the DNA-damage response (DDR), in which sensor proteins recognize the damage, remodel chromatin, and recruit specific kinases to coordinate cell cycle arrest, repair, or apoptosis and senescence if damage is irreparable [21,22,23,24,25]. Ataxia telangiectasia mutated (ATM) and ATM and Rad3-related (ATR) are the key kinases activated in the DDR for DSBs and SSBs, respectively. Poly [ADP-ribose] polymerase 1 (PARP1) recognizes SSBs and recruits X-ray repair cross-complementing protein 1 (XRCC1) and end-processing enzymes to restore continuity [26]. DSBs can be repaired through non-homologous end joining (NHEJ) or homologous recombination (HR) [26], linked to cell cycle control through the activation of TP53-binding protein 1 (53BP1) for NHEJ or breast cancer type 1 susceptibility protein (BRCA1) for HR. NHEJ is a quick and error-prone mechanism that operates throughout the cell cycle, particularly in G1, where the Ku70–Ku80 complex initiates repair, followed by DNA-dependent protein kinase (DNA-PK) activation and DNA end processing via Artemis and the MRE11–RAB50–NBS1 (MRN) complex. Instead, HR is restricted to S and G2 phases in which a homologous DNA template is available after replication. It begins with MRN complex binding, end resection, and formation of a DNA-repair protein RAD51 homologue 1 (RAD51) nucleoprotein filament, enabling strand invasion and accurate repair.

Considering the centrality of this pathway in the regulation of cell fate, the efficacy of radiotherapy is strictly linked to the capacity of cancer cells to detect and repair DNA damage. Cancer cells often have defective DDR pathways [27], making them more susceptible to damage from RT since persistent DSBs lead to mitotic catastrophe or apoptosis. However, tumors can alter this mechanism, enhancing specific and/or alternative repairs or circumventing DDR checkpoints, leading to radiation resistance. Genetic and epigenetic variability within tumors results in subpopulations with enhanced DDR or repair capacity. Overactivation of NHEJ [28,29] can promote this repair mechanism over HR, leading to worsening genomic aberrations. On the other hand, HR can be enhanced through the overexpression of RAD51 or BRCA1 [30,31]. Moreover, tumors often harbor mutations or alterations in checkpoint proteins, like p53 and CHK1/CHK2 (checkpoint kinase 1 and 2) [32,33], allowing cell cycle progression despite DNA damage. Alterations in DDR-associated factors pre- and post-treatment correlate with poor prognosis in cancer [34,35] and highlight the importance of modulating DDR to enhance radiation sensitivity, combining RT with chemotherapy and immunotherapy [36]. PARP inhibitors [37,38] block SSB repair, causing replication fork collapse and DSB accumulation, and are used to treat cancers with defective HR (e.g., BRCA mutations), such as breast, ovarian, head and neck, and non-small-cell lung cancers [39,40]. Checkpoint inhibitors impair cell cycle checkpoint regulation, targeting ATR, CHK1, and CHK2 [41,42]. Other strategies include the inhibition of heat-shock protein 90 (HSP90), which is involved in correcting the misfolding of DDR-related proteins: the HSP90 inhibitor ganetespib has been used in clinical trials as a radiosensitizer in rectal and esophageal cancers [43].

### 2.2. Apoptosis

Apoptosis is a programmed cell death that eliminates damaged cells and is a primary response to radiation-induced DNA damage. Dysregulation of apoptosis in cancer leads to survival and proliferation, contributing to radioresistance. RT activates apoptosis via both the intrinsic and extrinsic pathways [44]. The intrinsic pathway is triggered by stress signals (DNA breaks, ROS, endoplasmic reticulum stress) and p53 upregulation, enhancing pro-apoptotic BCL-2 homology 3 (BH3)-only proteins that neutralize anti-apoptotic B-cell lymphoma 2 (BCL-2) proteins. This allows the oligomerization of Bcl-2 homologous antagonist/killer (BAK) and Bcl-2 associated X protein (BAX), which permeabilize the mitochondrial membrane, leading to the release of cytochrome C and second mitochondria-derived activator of caspases (SMAC). Cytochrome C binds apoptotic protease-activating factor 1 (APAF1) to recruit and activate procaspase 9, forming the apoptosome, while SMAC antagonizes inhibitors of apoptosis proteins (IAPs). After its activation, caspase 9 cleaves and activates executioner caspases 3, 6, and 7, which perform the execution phase, consisting of cellular component degradation, cellular shrinkage, DNA fragmentation, membrane blebbing, and apoptotic body formation. Radiation can also induce the extrinsic pathway via the activation of the Fas death receptor, tumor necrosis factor (TNF) receptors, and TNF-related apoptosis-inducing ligand (TRAIL) receptors, leading to Fas-associated death domain (FADD) recruitment. FADD binds procaspase 8, forming the death-inducing signaling complex (DISC) in which procaspase 8 undergoes autocatalytic cleavage and activates the executioner caspases 3, 6, and 7 to perform apoptosis.

Tumors avoid apoptosis through the inactivation of p53 signaling, the overexpression of anti-apoptotic proteins, the downregulation of pro-apoptotic factors, the impairment of death-receptor signaling, the upregulation of IAPs, and the activation of survival pathways [45,46,47]. All these mechanisms can contribute to radiotherapy resistance. The therapeutic strategies often used in combination with radiotherapy to sensitize tumors with altered apoptosis include BH3 mimetics (e.g., venetoclax), p53 restoration through gene therapy, IAPs inhibitors, death receptor agonists, and survival-pathway blockers (e.g., everolimus). These strategies have demonstrated their efficacy in in vitro and/or in vivo studies for several types of tumors in combination with RT, such as lymphoma, head and neck cancer, colorectal cancer, gliomas, and breast cancer [48,49,50,51,52].

### 2.3. Hypoxia

Hypoxia is a condition characterized by low oxygen levels in tissues and is generated by an imbalance between tumor vascularization and high cellular demands. Hypoxia is a hallmark of solid tumors and is associated with increased resistance to chemotherapy and radiotherapy, genomic instability, and tumor invasion and metastasis [53]. Hypoxic cells adapt to survive in low-oxygen conditions, giving them a selective advantage. Low oxygen stabilizes the transcriptional hypoxia-inducible factors 1 alpha and 2 alpha (HIF-1α and HIF-2α), which enhance pathways essential for tumor growth and survival [54], such as angiogenesis, with the upregulation of vascular endothelial growth factor (VEGF) and metabolic shift toward glycolysis and lactate production (Warburg effect). Hypoxia also induces epithelial-to-mesenchymal transition (EMT) and matrix metalloproteinase expression, enhancing invasion and metastasis, while upregulating survival and proliferation genes. In addition, hypoxic niches promote immune evasion by recruiting immunosuppressive cells (regulatory T cells) and inhibiting cytotoxic T cells and natural killer (NK) cells. High levels of HIF-1α are considered a predictive biomarker for poor prognosis and response to chemotherapy and radiotherapy in cervical, breast, colorectal, and nasopharyngeal cancers [55,56,57,58].

Because oxygen amplifies ROS-mediated DNA damage, hypoxic regions are more resistant to radiation. Thus, targeting hypoxia is considered a promising strategy for developing novel cancer treatments. Currently used strategies include tumor oxygenation, oxygen-mimetic radiosensitizers (nimorazole and nitroimidazoles) [59,60], hypoxia-activated prodrugs (tirapazamine) [61] that selectively kill hypoxic cells, HIF signaling inhibitors (PX-478) [62], and VEGF inhibitors (e.g., bevacizumab) [63].

### 2.4. Metabolic Reprogramming

Tumor cells adapt to hypoxia, low pH, and nutrient deficiencies to promote survival and proliferation through metabolic reprogramming that drives radioresistance by supplying energy and suppressing immune responses. For energy demands, tumor cells utilize aerobic glycolysis rather than mitochondrial oxidative phosphorylation (Warburg effect), resulting in lactate accumulation. RT can enhance this mechanism, activating immunosuppressive pathways that facilitate tumor progression, recurrence, and resistance [64]. Moreover, cancer cells activate lipogenesis and mitochondrial fatty acid oxidation (FAO), producing acetyl-CoA that induces CD47 transcription to promote immunosuppression [65]. Combinations of FAO inhibitors and anti-CD47 antibodies can improve treatment after radiotherapy in glioblastoma [66]. Other metabolic changes involve the upregulation of glutamine synthetase and purine synthesis [67,68], which support tumor cell survival and DNA repair post-irradiation, contributing to radiation resistance.

Overall, metabolic reprogramming enables tumor cells to evade radiotherapy-induced cell death, and understanding these mechanisms is critical for developing combination therapies that target these pathways to improve RT outcomes. Combining metabolic inhibitors with immunotherapy and radiotherapy can overcome the suppressive tumor microenvironment and amplify radiosensitization [69].

### 2.5. Tumor Microenvironment

The tumor microenvironment (TME), the area in which the tumor is located, is characterized by hypoxia and low pH and comprises cancer-associated fibroblasts (CAFs), endothelial cells, tumor-associated macrophages (TAMs), and other immune cells [70]. Complex cross talk between cancer cells and the TME elements promotes growth, angiogenesis, invasion, and metastasis. Radiation can induce chronic inflammation, fibrosis, hypoxia, vascular damage, and immunosuppression in the TME [71]. Thus, a better understanding of the specific interactions between cancer cells, TME, and immune cells can help improve RT efficacy.

CAFs are activated by transforming growth factor beta (TGF-β) and secrete cytokines, chemokines, growth factors, and extracellular matrix (ECM) proteins [72] to favor tumor development. Moreover, CAFs recruit TAMs and establish an immunosuppressive condition in TME. Several factors expressed by CAFs after RT correlate with poor prognosis and participate in cell cycle arrest, DNA repair, ROS scavenging, and ECM remodeling, thereby mediating fibrosis, EMT, and treatment resistance [73,74,75]. While irradiation can increase the antitumoral immune response through the abscopal effect or the activation of the cyclic GMP-AMP synthase–stimulator of interferon genes (cGAS-STING) signaling pathway [76], it also activates immunosuppressive mechanisms in the context of the TME, triggering M2-type (pro-tumor) macrophage infiltration. TAMs are key players in innate immunity within the TME, driving tumor growth, angiogenesis, metastasis, and regrowth after chemotherapy or radiotherapy [77]. High levels of M2 TAM markers are associated with poorer survival rates and metastasis [78,79,80]. Ionizing radiation can also upregulate programmed death-ligand 1 (PD-L1) [81] and immunosuppressive chemokines [82], which promote the recruitment of regulatory T cells and M2 TAMs and prevent the activation of CD8+ cytotoxic T lymphocytes. These processes contribute to immune resistance and ultimately impair the effectiveness of RT.

A deeper understanding of these interactions can reveal targets and optimize RT–immunotherapy combinations to improve outcomes. Combining radiation with PD-L1 inhibitors [83], STING-activating drugs [84], and TGF-β inhibitors (bintrafusp alfa [85]) represents some promising therapeutic strategies aiming to modulate TME immunity and overcome radioresistance with a synergistic effect.

### 2.6. Cancer Stem Cells

Cancer stem cells (CSCs) are a small, but highly tumorigenic subpopulation of cancer cells with self-renewal, differentiation, and proliferation capacities that produce highly heterogeneous tumors [86]. CSCs occupy specific protective niches within the TME and are characterized by upregulated DNA repair, ROS scavenging, quiescence, and phenotypic plasticity that facilitates EMT. These properties are sustained by hypoxia and mutual signaling with the TME, contributing to immunosuppression and driving invasiveness and metastatic recurrence. Consequently, CSCs are more resistant to radiotherapy than normal tumor cells, and their activity is considered an adaptive response to the cytotoxic effects of irradiation [87]. CSCs limit radiation damage by activating checkpoint pathways that mediate cell cycle arrest and DNA repair, such as ATR-CHK1 and ATM-CHK2 signaling [88,89]. Hypoxia is a core regulator of CSCs, promoting the activation of survival pathways like Notch, TGF-β, sonic hedgehog, and the phosphoinositide 3-kinase/protein kinase B/mechanistic target of rapamycin signaling pathway (PI3K/AKT/mTOR) [90,91]. Furthermore, autophagy plays a relevant role in maintaining stemness phenotype and protecting from radiation through the Wnt/β-catenin signaling pathway [92]. Targeting CSC-specific pathways, autophagy, and the TME can offer potential strategies to reduce radiotherapy resistance and improve outcomes [93], but more studies are needed to understand the heterogeneous environment of cancer stem cells.

## 3. Proteomic Technologies and Applications in Cancer

In the last few years, proteomic applications in clinical contexts and cancer research have gained popularity as technology advances in MS-based proteomics. This is due to improvements in LC-MS instrumentation, with the high performance and automation of liquid chromatography separation (micro- and nanoflows) and the high sensitivity, resolution, scanning speed, and mass accuracy given by modern mass spectrometry analyzers (e.g., time of flight and Orbitrap). In addition, increasingly sophisticated software for data processing allows for confident and reproducible identification and quantification results. All these features enable the production of robust and high-throughput data that can detect and quantify the changes in protein profiles between different conditions (healthy vs. diseased, treated vs. control, responder vs. non-responder, etc.), allowing the identification of biomarkers and pathways for clinical applications.

### 3.1. Proteomic Workflow

A growing number of MS-based proteomic studies have been conducted to study the effect of radiotherapy and identify potential biomarkers associated with radiation resistance. The primary aim was to confidently detect and quantify proteins and proteoforms that change their abundance between radiosensitive and radioresistant samples, including an initial discovery phase and a validation phase to confirm protein identification and quantitative differences.

**Figure 2:**
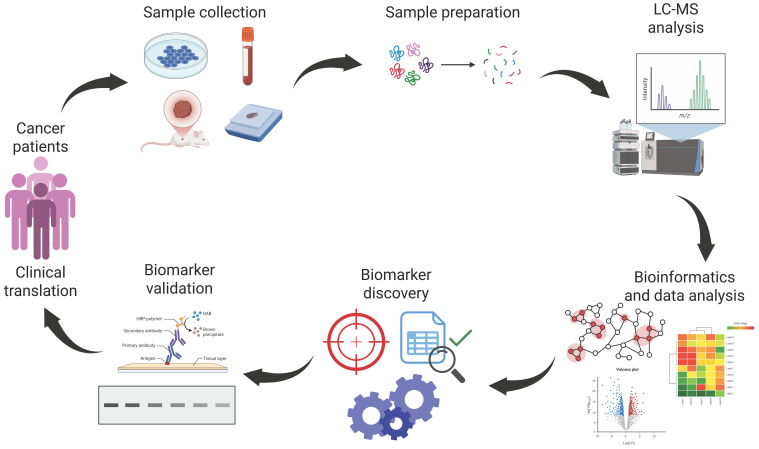
Workflow of bottom-up proteomics from sample collection to clinical translation. Created in BioRender. Perico, D. (2025) https://BioRender.com/obnbolk, accessed on 14 April 2025.

The typical approach applied in clinical proteomics (summarized in Figure 2) is a bottom-up “shotgun” strategy, in which the total protein content from biological samples is extracted and digested with trypsin, producing a peptide mixture that is analyzed by LC-MS/MS [94,95]. Various sample types can be studied, ranging from tissues (fresh frozen and formalin-fixed, paraffin-embedded—FFPE) to cell lines and biofluids (plasma, serum, or urine). Biofluid analysis is a trending topic in cancer proteomics due to its non-invasiveness and capacity to reflect systemic alterations [96]. Along with this, proteomic analysis on extracellular vesicles (EVs) extracted from cell culture media or biofluids [97] is emerging as a valuable tool to search for cancer biomarkers, since EVs represent a critical vehicle for cellular communication and can contain key signals responsible for cellular alterations. Other common samples used for proteomics in cancer and radiotherapy are xenograft models [98] in which human cancer cells or tumor tissues are implanted in immunocompromised mice. Xenografts represent in vivo models that mimic growth patterns, genetic features, and the microenvironment of human tumors, making them essential tools for studying tumor biology and evaluating therapeutic strategies.

After sample preparation, proteomic analysis is performed with micro- and nano-LC-high-resolution-MS/MS, which unites the high fractionation efficiency of HPLC systems (high yields and sensitivity using a very small amount of sample), with great performance in protein identification and quantification of mass spectrometry [99]. Specifically, high sensitivity, resolution, and mass accuracy are essential requirements to ensure reliable results, starting from full scan (precursor ions) and MS/MS scan (fragment ions) information, to rebuild the exact amino acidic sequence of peptides.

The LC separation step is typically based on a gradient of polar and organic solvents that elute the analyte mixture from a column in which the peptides are bound to the stationary phase depending on their physical–chemical properties, with reverse-phase chromatography used as the most common application [100]. The liquid flow containing the eluted peptides needs to be vaporized within an ion source to generate ions that can be analyzed by MS. The most used ion source in LC-MS analysis is electrospray ionization (ESI) [18], which is based on the generation of a spray through the application of high voltage, producing positively charged droplets at the capillary tip. As the droplets travel through the source, solvent evaporation occurs due to heat and nitrogen flow, shrinking the droplets until the charge repulsion overcomes the surface tension, thus releasing gas-phase ions of peptides. ESI is a soft ionization technique that gently ionizes molecules without fragmenting them and produces multi-charged state ions, allowing the detection of large molecules within the *m*/*z* range of mass spectrometers and subsequent fragmentation (MS/MS).

Then, the mass analyzer separates ions by measuring their mass-to-charge ratio (*m*/*z*). For protein analysis, quadrupoles, ion traps, time of flight (TOF), and Orbitrap are the most used analyzers and are often combined (Q–TOF, Q–Orbitrap, ion trap–Orbitrap) to perform tandem mass spectrometry in which the first analyzer selects and fragments precursor ions to be analyzed by the second one [101]. Each analyzer has its pros and cons regarding acquisition rate, sensitivity, resolution, mass accuracy, and dynamic range, but the sequential combination of different analyzers can highlight the strengths and limit the weaknesses. TOF measures the *m*/*z* of ions by determining their time to travel a fixed distance. Ions are accelerated by an electric field, with lighter ions reaching the detector faster than heavier ones. TOF analyzers offer high mass accuracy, broad mass range, and fast data acquisition, but typically have lower resolution and sensitivity than Orbitrap. Instead, an Orbitrap analyzer measures the *m*/*z* of ions based on their oscillatory motion around a central electrode. Ions are trapped in an electrostatic field, and their oscillatory frequencies are detected to determine their mass with exceptional accuracy and resolution. The major drawback compared to TOF is lower scanning speed.

The application of tandem mass spectrometry (MS/MS), in which the ions are selected and fragmented, enables the identification of molecular structures, the detection of specific functional groups, and the differentiation of closely related compounds. This is essential in proteomics to reconstruct the amino acid sequence of the peptides for correct protein identification. Typically, fragmentation occurs in a collision cell in which ions collide with neutral gas molecules (nitrogen, argon, or helium) in a process called collision-induced dissociation (CID) or high-energy collision dissociation (HCD), depending on the energy used [102]. This results in covalent bonds breaking at the peptide bond level, generating b- (N-terminus) and y- (C-terminus) ions in a fragmentation pattern.

Once the MS analysis is completed, the acquired spectra are processed with specific algorithms and search engines that perform database searches in which the experimental spectra are matched against theoretical ones derived from the in silico digestion of a protein sequence database of interest. Obtaining a confident list of identified proteins requires the application of scoring algorithms to evaluate the quality of the matches and statistics to control the false-discovery rate (FDR), usually with a target–decoy strategy [103]. The identification occurs at the peptide level, and protein identification is inferred using different modalities such as the parsimony principle (the smallest set of proteins that can explain all the identified peptides), minimum peptide rule (a minimum threshold of unique peptides belonging to a protein), protein grouping (isoforms grouped in protein families), and the use of unique and/or razor peptides (peptides that can map to multiple proteins, but are assigned to the protein with the most evidence) [104]. Modern platforms in MS-based proteomics are also able to perform peptide and protein quantification in order to extract upregulated or downregulated proteins useful for the discovery of potential biomarkers. Computational approaches for protein quantitation involve advanced algorithms and statistical methods to extract, normalize, and interpret peptide signals from mass spectra. Depending on the acquisition strategy, such as label-free quantitation (LFQ), isotopic labeling, or data-independent acquisition (DIA), computational pipelines map peptide intensities or spectral counts to corresponding proteins [105,106]. Plenty of tools are available to perform statistical and differential analysis to stratify samples (principal component analysis and clustering), study molecular mechanisms (functional enrichment analysis and identification of deregulated pathways), and investigate protein interactions (network analysis) with a systems biology approach [107].

The results obtained in the discovery phase of proteomic studies need to be validated to ensure their accuracy, reproducibility, and biological relevance so as to translate proteomic signatures into meaningful insights for biomedical research and clinical applications. This occurs with different orthogonal techniques such as enzyme-linked immunosorbent assay (ELISA), Western blotting, or targeted mass spectrometry [108] to confirm protein identification and quantification.

Of note, specific applications can deviate from the standard protocols, such as the study of post-translational modification (PTMs) like phosphorylation, acetylation, and glycosylation [109]. These modifications play key roles in regulating protein structure, localization, functions, and interactions, thus becoming important for biomarker discovery and disease mechanism studies. Proteomic investigation of PTMs requires specific sample preparation techniques to enrich modified peptides (e.g., using TiO_2_ for phosphopeptides) and different MS settings, such as diverse fragmentation techniques to preserve and detect labile PTMs (e.g., electron-transfer dissociation for phosphorylation). Moreover, PTM analysis is more challenging from a computational point of view, but several algorithms are available and capable of handling this task [110]. Specifically, it is necessary to manage large search spaces; differentiate true PTMs from noise, artifacts, or false positives; use scoring algorithms for site localization due to ambiguous mass shifts during fragmentation; and accurately quantify the coexisting modified and unmodified peptide forms.

Regardless of the proteomic pipeline used and the specific settings adopted in terms of LC separation, MS acquisition, and computational strategy, no method can analyze the whole proteome in a single experiment and retrieve all the biological information of a sample. For this reason, it is necessary to choose the method and application that can fit the objectives of the experiment the best, as well as employ complementary methodological approaches.

Moreover, emerging advancements are pushing these workflows even further. Single-cell proteomic platforms now quantify thousands of proteins per cell, uncovering intratumoral heterogeneity [111]. Trapped ion mobility with parallel accumulation–serial fragmentation (PASEF), coupled with microflow chromatography, dramatically increases peptide identifications and throughput in minutes per sample [112]. Library-free DIA tools leverage deep-learning spectral prediction to boost sensitivity, reproducibility, and data completeness [113]. Spatial proteomics using MALDI (matrix-assisted laser desorption/ionization) imaging maps protein landscapes within hypoxic, fibrotic, or stem-cell niches in tumor sections, revealing microenvironmental drivers of resistance [114]. Finally, top-down proteomics directly profiles intact proteoforms, capturing critical PTMs and splice variants [115].

**Figure 3:**
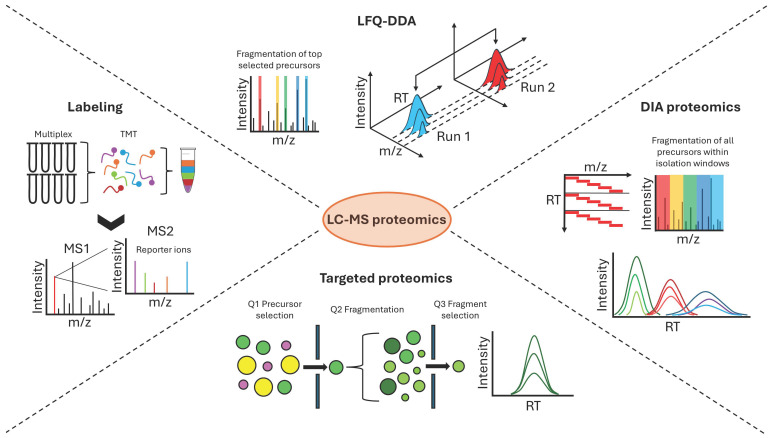
Main techniques used in LC-MS proteomics for cancer research. MS1: first stage MS; MS2: tandem MS; RT: retention time; TMT: tandem mass tag; LFQ: label-free quantification; DDA: data-dependent acquisition; DIA: data-independent acquisition; Q: quadrupole.

Among the several proteomic approaches, the most used in cancer and radiotherapy studies are label-based techniques, label-free data-dependent acquisition, data-independent acquisition, and targeted proteomics (Figure 3).

### 3.2. Label-Based Quantitative Proteomics

Label-based quantitative proteomics refers to techniques that use stable isotopic or chemical labels to measure protein abundance in complex biological samples [116]. These methods enhance the precision and reproducibility of quantitation in proteomic studies, particularly in comparative analyses, including cancer applications. Labeling techniques enable relative and absolute quantitation, the latter using internal standards. The main methods are isobaric tags for relative and absolute quantification (iTRAQ) [117] and tandem mass tags (TMTs) [118]. These approaches utilize isobaric reagents to label primary amines of peptides, allowing the simultaneous comparison of up to 8 samples (8-plex for iTRAQ) or 16 samples (16-plex for TMT) that are pooled and analyzed in a single LC-MS run. The iTRAQ and TMT reagents consist of a reactive group that binds the primary amines, a balance group that makes the labeled peptides from each sample isobaric, and a unique reporter group that is released upon fragmentation and used for quantification (reporter ion intensities). The main advantages of these techniques are multiplexing capabilities, high sensitivity, and high accuracy and reproducibility in quantitation due to internal sample normalization that minimizes experimental variations. This enhances the reliability of quantitative proteomics and enables robust statistical analysis, especially for large-scale proteomic studies. Conversely, the disadvantages include the complex and time-consuming sample preparation, high reagent cost, incomplete labeling, potential interference at the reporter ion level in complex samples, and ratio compression due to the co-elution of peptides. Another widespread labeling technique is SILAC (stable isotope labeling by amino acids in cell culture), which is based on the metabolic incorporation of isotopically enriched amino acids as cells grow. The quantitation is based on the comparison between the intensities of “light” (natural) and “heavy” (labeled) peptides, giving high accuracy and multiplexing capacity. The main drawback is the restriction to cell cultures [119].

### 3.3. Label-Free Quantification with Data-Dependent and Data-Independent Acquisition

Label-free quantification (LFQ) is a widely used approach in quantitative proteomics by LC-MS, in which the relative abundance of proteins is measured across different biological samples without any labeling [120]. This can be based on spectral counting (quantitation at MS2 level) or peptide ion intensity (quantitation at precursor level). Spectral counting consists of counting and comparing the number of identified MS/MS spectra for the peptides of a protein and relies on the empirical observation that the number of fragmentation spectra of a peptide are proportional to the amount of the corresponding protein in the sample, allowing a relative quantification [121]. On the other hand, the peptide ion intensity approach is based on the measurement of the chromatographic peak area of precursor ions, which linearly correlates with peptide abundance [122]. MS/MS spectrum information is used for peptide identification, while the mass peak at the precursor level is visualized as a function of the LC retention time with an extracted ion chromatogram (XIC) in which the area under the curve (AUC) is integrated to obtain the peptide abundance. Spectral counting is a simple and robust method that requires minimal data processing, but results in bias towards highly abundant proteins, saturation issues due to overrepresented proteins, and lower sensitivity and quantitation precision compared to peptide intensity. The latter offers a broader dynamic range, higher sensitivity and precision, and more accurate and linear quantification than spectral counting. The downsides are related to data complexity, high dependence on instrumentation performance, co-eluting peptides that can interfere with quantitation, and more computational needs for raw LC-MS processing to perform feature detection, retention time alignment, peak matching, normalization, and peptide-to-protein roll-up quantification.

Compared to labeling techniques, the benefits of LFQ include a more straightforward process, broad applicability for diverse sample types and purposes, scalability for large studies and complex experimental designs, and lower cost. The major drawbacks are lower accuracy and precision due to higher technical variability in sample preparation and susceptibility to instrument fluctuations (batch effect), as well as challenges for quantifying low-abundance proteins and data processing since sophisticated algorithms and tools are required for peak alignment and normalization.

Regarding MS acquisition settings, LFQ can be conducted with two different strategies: data-dependent (DDA) and data-independent (DIA) acquisition. In DDA [123], the mass spectrometer alternates between MS1 and MS2 scans so that it can detect all precursor ions and select the most intense ones (top N strategy) for fragmentation. The selection is dynamically influenced by peptide abundance in the sample, and an exclusion time is set to avoid the repeated selection of the same ion and to pick less intense signals. In DIA [124], the entire mass range of interest is divided into isolation windows of predefined sizes (fixed or variable depending on the ion density along the mass range), and all ions detected within each window are simultaneously fragmented and analyzed. DDA ensures high sensitivity and specificity, an efficient dynamic range, and robust data analysis that relies on established pipelines for identification, quantification, and statistical analysis (e.g., Proteome Discoverer by Thermo Fisher Scientific, MaxQuant, FragPipe, OpenMS) [125]. DDA suffers from limited reproducibility, undersampling of low-abundance peptides, bias towards highly abundant peptides, and the presence of missing values across samples due to stochasticity in ion selection. DIA overcomes most of the DDA limitations, providing higher reproducibility, consistent and comprehensive data collection, and improved quantification accuracy due to data completeness. DIA issues involve reduced specificity and higher data complexity due to co-isolation and co-fragmentation of ions that can impair identification. Moreover, the complexity of spectra is challenging from a computational point of view and requires dedicated platforms. Traditionally, DIA employed spectral libraries generated from DDA runs [126] to extract all the information for protein identification. This method gained popularity due to high-confidence quantification, but it can be challenging and time-consuming. More recently, software developed for DIA proteomics (e.g., Spectronaut by Biognosys, DIA-NN, FragPipe, Skyline) implemented the so-called library-free approach [127], in which the identification is performed directly from DIA data, and powerful algorithms make in silico predictions of peptide fragmentation patterns and retention times from protein sequence databases. Advancements in DIA proteomics are making these platforms increasingly reliable for protein quantification and the handling of high-throughput data for large-scale studies.

### 3.4. Targeted Proteomics

The methods described above are related to an untargeted approach to MS-based proteomics, which aims to identify as many proteins as possible for discovery studies. In contrast, targeted proteomics selectively quantifies specific peptides or proteins in complex biological samples [128]. This quantitative approach is characterized by high specificity and sensitivity (efficient quantitation of low-abundance proteins with reduced background noise), high reproducibility, and accurate quantification, providing both absolute and relative quantitation in a label-based or label-free manner. Moreover, by focusing only on specific targets of interest, data acquisition is faster, less complex, and with fewer false positives than untargeted methods. One of the most important features of targeted proteomics is the possibility of bridging discovery and validation analyses [129], which is essential for precision medicine and helps fill the gap between molecular analysis and clinical applications. A remarkable strategy can be the use of untargeted proteomics for biomarkers, pathways, and mechanisms discovery, to be validated using targeted approaches. Limitations of targeted proteomics include prior knowledge of targets, limited throughput, and complex assay development that requires an extensive optimization of MS settings.

The main methods used in targeted approaches are selected reaction monitoring (SRM), multiple reaction monitoring (MRM), and parallel reaction monitoring (PRM) [130]. SRM is the simplest method, and monitors a specific precursor ion and its fragment ions in a triple-quadrupole mass spectrometer. The first quadrupole selects the precursor ion of interest, the second one fragments it, and the third one selects a specific transition for detection. MRM is an advanced form of SRM that monitors multiple precursor–fragment transitions simultaneously, and each peptide is identified by unique transition pairs (precursor and fragment). This method improves throughput and confidence in quantification compared to SRM. PRM is a targeted method that uses mass spectrometers with high resolution and mass accuracy, like Orbitrap and Q-TOF. After the precursor ion selection, all the corresponding fragment ions are collected and detected in parallel in the high-resolution analyzer (Orbitrap or TOF). This strategy provides higher specificity and confidence than MRM due to high-resolution transition detection and does not require fragment ion preselection.

## 4. Identification of Radiotherapy Resistance Biomarkers by Proteomics

Many potential biomarkers of radioresistance and radiosensitivity have been identified through proteomic approaches applied in in vitro, in vivo, and clinical studies. Despite the importance of this research in unveiling resistance mechanisms and new therapeutic targets, the translation of protein biomarkers into clinical practice is still limited and more efforts are needed to confirm proteomic results. In this section, proteomic studies applied to radioresistance biomarker discovery in different types of cancer are summarized, and the list of biomarkers with related information is reported in Table 1.

It should be noted that in some clinical studies presented here, proteomic biomarkers of radioresistance were identified either by comparing pretreatment specimens from patients who ultimately responded to radiotherapy versus those who did not or by comparing paired pre- versus post-treatment samples. Although these approaches offer clinically relevant signatures, the extent to which these markers reflect true radioresistant phenotypes remains to be validated in recurrence-derived materials, and future proteomic analyses of radiorecurrent lesions will be essential to bridge this gap.

### 4.1. Head and Neck Cancers

Head and neck cancer (HNC) includes a group of malignancies affecting the oral cavity, larynx, pharynx, nasal cavity, paranasal sinuses, and salivary glands, and the most common type is squamous cell carcinoma. HNC is the sixth-leading cancer by incidence and the eighth by death worldwide. Despite the efficacy of RT as the primary treatment of HNC (5-year survival rates of 40%–50%), tumor recurrence caused by radioresistance remains an unresolved issue [131].

In the past, most studies were focused on analyzing radioresistant (RR) cell lines compared to sensitive ones and applying gel-based proteomics with 2D electrophoresis coupled with MALDI-TOF mass spectrometry [132,133]. This technique was widespread in protein analysis before the establishment of gel-free LC-based proteomics, which offers superior results in terms of yield, sensitivity, precision, and throughput.

More recently, studies applying LC-MS methods have emerged to research RT-resistance biomarkers in HNC. Specifically, labeling techniques such as iTRAQ and TMTs were used to analyze nasopharyngeal carcinoma (NPC) cell lines. Chen et al. performed the proteomic analysis of CNE2-RR cells secretome and reported CD166, cofilin 2 (CFL2), fibrillin 2 (FBN2), and sulfhydryl oxidase 1 (QSOX1) as potential markers involved in RT resistance due to their role in cell adhesion, migration, and invasion contributing to tumor cell plasticity, EMT, and metastasis [134]. Similarly, another study identified and validated mitogen-activated protein kinase 15 (MAPK15) as a regulator of radioresistance by attenuating ROS accumulation and promoting DNA repair [135]. Knockdown of MAPK15 impaired clonogenic survival, decreased cell viability, and increased apoptosis following exposure to irradiation, while its upregulation promoted cell survival and reduced apoptosis in NPC cell lines.

Regarding clinical samples, Zhang et al. performed label-based quantitative proteomics (TMTs) on sera of 44 NPC patients, identifying serpin family D member 1 (SERPIND1), complement C4B, secreted protein acidic and rich in cysteine (SPARC), peptidyl-prolyl cis-trans isomerase B (PPIB), and family with sequence similarity 173 member A (FAM173A) as proteins able to discriminate between radiosensitive and radioresistant groups [136]. SERPIND1 is a serine protease inhibitor involved in coagulation. Although its precise role in radioresistance remains unclear, its upregulation in NPC patients suggests a potential role in regulating the TME, possibly through angiogenesis [136]. C4B is a component of the classical complement pathway, which is typically dysregulated in cancers and can contribute to the immunosuppressive environment within the TME [137]. SPARC, a modulator of the extracellular matrix, can influence the tumor microenvironment and EMT [138], facilitating tumor growth and invasion. PPIB has a role in protein folding and can induce resistance by enhancing DNA repair and regulating hypoxia and endoplasmic reticulum (ER) stress, which is a hallmark of cancer [139]. FAM173A is a mitochondrial protein with limited characterization in cancer. Its downregulation may indicate a role in mitochondrial dysfunction and oxidative stress regulation [140]. Another study performed on FFPE tissues from 18 patients with locally advanced oral squamous cell carcinoma (OSCC) treated with neoadjuvant radiotherapy identified and validated galectin 7 (LGALS7) as a predictor of RT resistance with high specificity and sensitivity, as suggested by 5-year disease-specific survival rates that significantly differed between low- and high-abundance groups of galectin-7 [141]. Galectin 7 is a beta-galactoside-binding lectin that plays a significant role in various cellular processes, including cell adhesion, apoptosis, inflammation, and immune response, and its dysregulation can lead to resistance through anti-apoptotic effects and immune evasion [142].

Jackson et al. reported the proteomic analysis of 124 FFPE tissues of human papillomavirus-associated oropharyngeal squamous cell carcinoma (HPV+ OPSCC) with and without recurrence. Using DIA-MS, the authors identified a signature of 26 deregulated proteins that were associated with recurrence-free survival and were able to stratify patients based on recurrence risk. Among these proteins, keratin type I cytoskeletal 17 (KRT17), 72 kDa type IV collagenase (MMP2), protein S100A4, and lamin B1 (LMNB1) were upregulated, while phosphatidate cytidylyltransferase 2 (CDS2), GTPase-activating protein and VPS9 domain-containing protein 1 (GAPVD1), and WD repeat-containing protein 81 (WDR81) were downregulated [143]. KRT17 is a cytoskeletal protein whose overexpression has been associated with poor prognosis in multiple cancers, with implications in immune evasion [143]. MMP2 is an ECM-degrading enzyme involved in tumor invasion, EMT, and angiogenesis [143]. Similarly, S100A4, a multifunctional calcium-binding protein involved in cell motility, invasion, and metastasis, is a well-established promoter of EMT, and its upregulation facilitates resistance and recurrence [144]. LMNB1 is a component of the nuclear envelope involved in chromatin remodeling, and its increase may reflect a protective role in maintaining nuclear integrity and avoiding apoptosis in response to radiation-induced DNA damage [143]. CDS2 is involved in phospholipid biosynthesis, possibly linked to metabolic reprogramming [145]. GAPVD1 plays a role in vesicle transport, possibly promoting proliferation and survival via altered intracellular trafficking [146]. WDR81 is associated with protein degradation and autophagy, and its downregulation can alter cellular stress responses or enhance survival under radiation by inhibiting autophagic cell death [147].

Additionally, a recent work in which the authors used an integrated multi-omics approach in analyzing the tissues of recurrent OSCC patients revealed that Ras-related protein RRAS may serve as a potential predictor of recurrence and resistance. RRAS activates a signaling pathway critical for cell survival and proliferation, promoting tumor progression and metastasis [148].

Together, these studies indicate that various proteins regulate radiation sensitivity in HNC and may be utilized to predict RT effectiveness and support the development of new anticancer treatment strategies.

### 4.2. Breast Cancer

Breast cancer (BC) is the most prevalent and commonly diagnosed malignancy among women, being one of the leading causes of cancer-related deaths. RT is a standard component of breast cancer treatment, often alongside surgery and chemotherapy; however, metastasis and recurrence are widely spread, limiting RT efficacy. Moreover, BC is a highly heterogeneous disease characterized by different subtypes, such as estrogen receptor (ER+), progesterone receptor (PR+), human epidermal growth factor 2 (HER2+), and triple-negative, that can respond differently to treatments [149]. Thus, discovering resistance biomarkers is essential to improve BC therapies.

Kim et al. used the SILAC method to investigate the proteomic profiles of MDA-MB-231 BC cells treated with single or fractionated RT and found several tumor-associated factors, such as cathepsin D (CTSD), gelsolin (GSN), and mannose receptor C type 2 (MRC2), upregulated after irradiation and in a dose-dependent manner [150]. CTSD is an aspartic protease involved in protein degradation, apoptosis, and inflammatory response. GSN is an actin-binding protein that regulates cytoskeletal dynamics, cell motility, and innate immunity. MRC2 is a lectin receptor involved in ECM remodeling and immune cell motility. The results suggested the potential role of these proteins in cancer progression and immune modulation during radiotherapy.

Two studies applied label-based and targeted proteomics (MRM and PRM) to study the protein kinase profiles (kinome) of radioresistant BC cells MCF-7 [151,152]. The authors selected and validated checkpoint kinase 1 (CHK1), cyclin-dependent kinases 1 and 2 (CDK1 and CDK2), and TATA-box binding protein-associated factor 9 (TAF9) as proteins increased in RR cells, suggesting an important role of DNA repair, cell cycle, and transcription regulation in BC radioresistance.

Another study employed SILAC-MS to study the phosphoproteome of irradiated cells with and without fisetin treatment. Fisetin is a plant flavonoid with a radiosensitization effect, since it can block DNA repair mechanisms by interfering with ribosomal S6 kinase (RSK) and AKT signaling, leading to the inhibition of Y box-binding protein 1 (YB-1) phosphorylation [153]. The phosphoproteomic approach revealed downregulated phosphosites in proteins involved in DNA repair, such as protein DEK, nucleolin (NCL), XRCC1, and DNA topoisomerase 2 alpha (TOP2A), suggesting their role in regulating DDR.

Notably, one study applied proteomics to investigate the role of cancer stem cells in radioresistance. Lamb et al. explored the potential of doxycycline as a radiosensitizer by targeting tumor-initiating cells. Using LFQ DDA proteomics, the authors found that DNA-dependent protein kinase (DNA-PK or PRKDC) was upregulated in mammospheres derived from BC cells, helping to maintain stem-like properties, and became significantly reduced when cells were treated with doxycycline, reducing mammosphere-formation activity [154]. DNA-PK is an enzyme essential in DSBs repair and maintaining mitochondrial DNA integrity, thus regulating mitochondrial metabolic functions.

Of note, Thomas et al. evaluated the potential of studying extracellular vesicle (EV) proteomic profiles as biomarker sources for radiotherapy resistance in breast cancer. Since hypoxia can increase the release of EVs in the TME to support invasion and survival, EVs can represent a valuable tool for characterizing hypoxia-induced radiation resistance. Preliminary results of SILAC performed on SKBR3 cells indicated that extracellular signal-regulated kinase 2 (ERK2) and glycogen synthase kinase 3 alpha and beta (GSK3A and GSK3B), involved in cell proliferation, survival, and apoptosis signaling pathways, were upregulated under hypoxic conditions, suggesting the role of EV cargos in mediating radioresistance [155].

Proteomic analysis was also applied to xenograft models to study radioresistance. Yadav et al. used LFQ DDA to analyze breast cancer xenografts from mice treated with RR and normal cells. Based on pathway analysis, metabolism (mainly glycolysis and lipid-related), protein folding, cytoskeleton organization, and ribosome biogenesis were among the most deregulated, though no protein was further validated [156].

Overall, these findings can reveal potential targets for enhancing radiation sensitivity and identifying biomarkers to predict radiation response in BC.

### 4.3. Lung Cancer

Lung cancer is one of the deadliest and most common cancers worldwide. The main types of lung cancer are small-cell lung carcinoma (SCLC) and non-small-cell lung carcinoma (NSCLC), with different prevalence, growth, and spread features, diagnosis stages, prognosis, and treatment options [157]. Radiotherapy is a key adjuvant treatment for NSCLC patients who are not eligible for surgery, but often the tumor develops resistance. Identifying potential biomarkers for radiation resistance is gaining importance for predictive and therapeutic applications in NSCLC.

Some studies have compared protein profiles of radiosensitive and radioresistant cell lines to investigate molecular changes. Pan et al. analyzed RR H2170 cells with TMT-based proteomics, identifying 8 upregulated and 19 downregulated proteins involved mainly in DNA repair, cell adhesion, cell migration, and ECM remodeling. Specifically, methyl-CpG-binding domain protein 4 (MBD4), involved in DNA repair, metalloproteinase inhibitor 3 (TIMP3), and podocalyxin (PODXL), both related to cell motility and invasion, were upregulated in RR cells [158]. Another study focused on analyzing proteomic and lysine 2-hydroxybutyrylation (Khib) profiles of RR cells. This PTM is an acetylation modification that was previously associated with cancer progression and metastasis, with roles in regulating metabolic processes such as glycolysis, TCA cycle, and fatty acid oxidation. Among the deregulated proteins identified, epidermal growth factor receptor (EGFR) was upregulated in RR cells, both for protein and Khib levels, while protein kinase C alpha (PRKCA) was downregulated. EGFR was validated as a biomarker for radiation resistance because of its role in activating cell proliferation, survival, invasion, and angiogenesis [159]. PRKCA is a serine/threonine kinase involved in proliferation, survival, and migration signaling pathways, indicating a potential role in therapy resistance [160]. Li et al. used a combined transcriptomic and proteomic approach (TMT labeling) on irradiated cells, identifying fibronectin 1 (FN1) and thrombospondin 1 (THBS1) as potential predictive biomarkers of RT response, since their high levels were associated with low survival rates [161]. FN1 and THBS1 are glycoproteins involved in ECM-receptor signaling pathways, regulating cell adhesion, migration, survival, tissue remodeling, and immune response. Their increase has been associated with tumor progression and therapy resistance via TME modulation and immune evasion.

Yan et al. explored the effect of the natural compound tanshinone I as a radiosensitizer in lung cancer cells. Proteomic analysis using SILAC showed the downregulation of the pro-oncogenic protein phosphoribosyl pyrophosphate aminotransferase (PPAT) in RR cells treated with tanshinone I, which can serve as a potential PPAT inhibitor [162]. PPAT is a key enzyme in the de novo purine nucleotide biosynthetic pathway, which is typically overexpressed in cancer, contributing to cell proliferation and invasion.

Moreover, a proteomic analysis conducted on the secretome of irradiated CAFs derived from NSCLC patients revealed the presence of proteins with immunomodulatory function, highlighting the importance of CAF signaling in regulating inflammatory response and immunosuppression after irradiation [163].

Concerning clinical applications, Huang et al. analyzed proteomic profiles of sera comparing sensitive and resistant groups to chemoradiotherapy. Using LFQ DDA, alpha-1-antitrypsin (SERPINA1) was identified as a potential biomarker for the resistant group and was further validated by ELISA [164]. SERPINA1 is a serine protease inhibitor with roles in inflammatory responses, indicating an immunomodulatory function that can promote survival and immune evasion [165]. Walker et al. performed iTRAQ proteomics on plasma from patients with locally advanced NSCLC treated with RT. Proteomic profiles revealed that C-reactive protein (CRP) and leucine-rich alpha-2-glycoprotein 1 (LRG1) were significantly upregulated in patients with reduced survival time, suggesting their role as predictive markers of survival and prognosis [166]. CRP is a key protein of the acute-phase response involved in inflammation and contributing to a pro-metastatic TME, while LRG1 can promote neovascularization under hypoxia by activating TGF-β signaling, thus supporting tumor growth. The inhibition of LRG1 can represent a therapeutic strategy to regulate angiogenesis [166].

In summary, proteomics has provided several insights regarding pathways and biomarkers associated with RT resistance in lung cancer.

### 4.4. Prostate Cancer

Prostate cancer is the most common tumor in men worldwide and can be treated with different options based on stage and aggressiveness. RT is often used as primary treatment for localized cancer, typically along with hormone therapy (androgen deprivation), or as adjuvant therapy after surgery. Despite significant improvements in radiation delivery techniques, approximately 50% of prostate cancer patients treated with RT suffer from recurrence within five years [167]. Personalized approaches are needed to stratify patients based on RT responsiveness and to evaluate alternative or combinatory therapies. Identifying radioresistance biomarkers allows the optimization of treatment strategies, minimizing side effects.

Chang et al. performed a proteomic analysis by LFQ DDA of three prostate cell lines, comparing radiation-sensitive and radiation-resistant ones. Fructose-bisphosphate aldolase A (ALDOA), alpha-2-HS-glycoprotein (AHSG), vimentin (VIM), 14-3-3 protein epsilon (YWHAE), and peroxiredoxin 6 (PRDX6) were identified as upregulated in RR cells. ALDOA was further validated by WB and IHC in cells and xenografts and functionally verified through small interfering RNA (siRNA) knockdown to induce radiosensitivity. ALDOA is a glycolytic enzyme associated with cancer progression and metastasis due to metabolic reprogramming, and these results highlighted its potential as a novel therapeutic target in combination with RT [168]. AHSG is a serum glycoprotein implicated in inflammation and tumor progression [169], while VIM is a cytoskeletal protein and a marker of EMT associated with invasiveness [168]. YWHAE plays a role in signal transduction, cell cycle regulation, and stress response, helping in maintaining cellular homeostasis under stress conditions. Similarly, PRDX6 is involved in oxidative stress regulation, scavenging ROS that can be produced upon irradiation [168].

Kurganovs et al. analyzed protein profiles of RR DU145 prostate cancer cells and found several deregulated pathways related to radiation resistance, such as glycolysis, hypoxia, DNA repair, and EMT. Specifically, CD44, a cell surface glycoprotein involved in cell adhesion and migration, was upregulated in RR cells and was suggested as a driver of cancer progression and therapeutic resistance, making it a potential therapeutic target [170].

Another study performed a multi-omics analysis (transcriptome, methylome, and proteome) on a prostate cancer cell model resistant to DNA damage to explore the adaptation mechanisms. DIA proteomic results showed an upregulation of cell adhesion and unfolded protein response (UPR) and a downregulation of DNA repair following irradiation. Asparagine synthetase (ASNS), an enzyme responsible for converting aspartate and glutamine into asparagine and glutamate, was upregulated in response to DNA damage and RT by different omics profiling: its role in ER stress suggested the centrality of UPR in mediating resistance mechanisms [171].

Moreover, Hao et al. revealed that lactate dehydrogenase A (LDHA) and the glycolytic pathway were increased in a radiation-resistant xenograft mouse model. Knockdown of LDHA with siRNA and inhibition of enzymatic activity with a specific inhibitor showed radiosensitization via reduced hypoxia, EMT, DNA repair and autophagy, and increased DNA damage and apoptosis [172].

Other studies were focused on the proteomic analysis of tissues. Keam et al. used DIA proteomics to analyze eight patients pre- and post-irradiation, divided into discovery and validation cohorts (fresh and FFPE tissues, respectively). The results showed an enrichment of immune signaling and complement cascade, and a core of 49 radiation-responsive candidates was extracted, including proteins involved in immune evasion, TME modulation, inflammatory response, and oxidative stress regulation [173]. Among them, ferritin light chain (FTL) and fibrinogen gamma chain (FGG) were upregulated, while prostatic acid phosphatase (ACPP) was downregulated. FTL is a ferritin subunit that exerts a crucial role in iron homeostasis; its contribution to radiation resistance can be related to ROS scavenging and EMT [174]. FGG is part of fibrinogen, a key component for blood coagulation and wound healing. Its upregulation can be associated with tissue remodeling, angiogenesis, and inflammation, contributing to cancer progression [175]. ACPP is a tyrosine phosphatase mainly produced by prostate epithelial cells and typically serves as a diagnostic and prognostic biomarker for prostate cancer. Nevertheless, intracellular ACPP is involved in cell growth signaling, and reduced levels were associated with tumor progression and castration-resistance development [176].

Furthermore, Williams et al. explored the proteomic and transcriptomic landscape of a multiresistant neuroendocrine prostate cancer case study. Four tissue sections were analyzed, corresponding to four different stages: at diagnosis, pre-irradiation, post-irradiation, and at recurrence. The overlapping of transcriptomic and proteomic results showed 11 upregulated and 8 downregulated proteins at recurrence [177]. Among the upregulated proteins, DNA topoisomerase 2 alpha (TOP2A) is an enzyme that maintains DNA topology, regulating DNA replication and chromosome segregation. It has already been reported as a prognostic biomarker for aggressive and metastatic prostate cancer subtypes. Calcium-binding proteins S100A8 and S100A9 are involved in inflammation and immune response, thus contributing to an immunosuppressive tumor microenvironment, as well as facilitating cancer cell invasion. Karyopherin alpha 2 (KPNA2) regulates protein import into the nucleus, and high levels have been linked to prostate cancer progression and recurrence via EMT regulation. Spondin 2 (SPON2) is an extracellular matrix protein proposed as a serum biomarker for prostate cancer and implicated in cell adhesion and innate immunity [177]. Regarding the downregulated proteins, anterior gradient protein 2 homologue (AGR2) is a protein disulfide isomerase localized in the ER, with roles in protein folding and stress response. Its lower abundance has been associated with metastasis and can be predictive of prostate cancer recurrence. Multidrug resistance-associated protein 4 (ABCC4), an ATP-binding cassette transporter, and arachidonate 15-lipoxygenase B (ALOX15B), an enzyme involved in lipid metabolism and inflammation, have been known to decrease in prostate cancer and with tumor progression [177].

Taken together, these studies have revealed several potential biomarkers and highlighted the importance of proteomics for explaining prostate cancer radiation resistance.

### 4.5. Other Cancers

Radiotherapy is also a valid treatment option for other types of cancer, and the search for biomarkers by proteomics can give clues regarding radiation resistance mechanisms.

Gao et al. performed TMT-based quantitative proteomics on RR esophageal squamous cell carcinoma (ESCC) cell lines. Protein S100A6, protein-glutamine gamma-glutamyltransferase 2 (TGM2), and glycogen phosphorylase, brain form (PYGB) were upregulated in RR cells compared to sensitive ones and were selected as potential biomarkers. Analyses with siRNA showed that the knockdown of these proteins induced radiosensitization by promoting DNA damage after irradiation, suggesting their involvement in DSB repair [178]. S100A6 is a cellular calcium signaling modulator and its upregulation post-irradiation has been previously reported for other cancer cells, typically associated with EMT. Additionally, immunohistochemistry (IHC) conducted on primary and recurrent tumor tissues revealed a positive correlation between S100A6 protein level and recurrence. TGM2 is a multifunctional enzyme with ATPase, GTPase, kinase, and protein disulfide isomerase activity, and is thus involved in several cellular processes. Despite its role in ESCC not yet having been defined, it has been reported that TGM2 can support radiation resistance by regulating autophagosomes and lysosomes fusion in glioblastoma [179]. PYGB, an enzyme involved in glycogen metabolism, has previously been associated with cancer progression and metastasis [180].

Yu et al. explored the potential of vitamin D as a radiosensitizer in colorectal cancer (CRC) cells. Proteomic analysis with LFQ-DDA showed the upregulation of cystatin-D (CST5) and plasminogen activator inhibitor 1 (PAI1) in irradiated cells treated with vitamin D. Among the identified deregulated pathways, cell migration, cell motility, and JAK/STAT3 signaling were the most enriched. The results suggested that the increase of these proteins inhibited EMT via blocking Janus kinase/signal transducer and activator of transcription 3 (JAK/STAT3) signaling, leading to radiosensitization [181]. CST5 is a cysteine protease inhibitor with a role in suppressing cell migration in CRC. PAI1 is the inhibitor of urokinase-type plasminogen activator, which exerts a crucial role in cancer progression and metastasis. Moreover, PAI1 can suppress tumor migration by blocking the binding site between integrin and vitronectin.

Another study proposed a mechanism of radioresistance based on Ras-related protein Rab-5C (RAB5C) action. Proteomic analysis of tissues from rectal cancer patients treated with neoadjuvant chemoradiotherapy revealed the upregulation of RAB5C and X-ray repair cross-complementing proteins 5 and 6 (XRCC5 and XRCC6, also known as Ku70 and Ku80), confirmed also in an in vitro model using irradiated cells. RAB5C is a key regulator of membrane trafficking and endocytosis, while Ku70/80 represents a heterodimer involved in DSB repair with NHEJ. Immunofluorescence analysis conducted on cells showed that irradiation induced EGFR internalization and localization to the nucleus by RAB5C activity. This enhanced Ku70/80 level and DNA repair, leading to radioresistance [182].

Other studies applied proteomics to examine glioblastoma radiation resistance. Glioblastoma is the most common and aggressive brain tumor, and originates from astrocytes in the glia. Despite the use of multimodal therapy, with a combination of RT and chemotherapy (i.e., temozolomide) following surgery, most patients experience recurrence, and the prognosis remains poor [183].

Rajendra et al. analyzed glioblastoma RR cells with iTRAQ proteomics, finding an upregulation of proteasome components. Specifically, proteasome activator complex subunit 1 (PSME1), proteasome subunit alpha type 7 (PSMA7), and proteasome subunit beta type 4 (PSMB4) were increased in RR cells and were further validated by Western blot. The enhanced proteasomal activity regulated cell survival by triggering nuclear factor kappa-light-chain-enhancer of activated B cells (NF-kB) signaling and the pharmacological inhibition of proteasome reduced NF-kB transcriptional activity, leading to radiosensitization [184].

Clavreul et al. used DIA proteomics to analyze 50 tissue and 30 serum samples from glioblastoma patients treated with chemoradiotherapy, comparing short-term and long-term survivors. Pathway analysis showed that the deregulated functions were related to ROS detoxification. Malate dehydrogenase 1 (MDH1) and ribonuclease inhibitor 1 (RNH1) were increased in the serum of short-term survivors. Additionally, fatty acid-binding protein 7 (FABP7) was decreased in tissues of short-term survivors and validated by IHC [185]. MDH1 is an enzyme essential for energetic metabolism, operating in the malate-aspartate shuttle to maintain redox homeostasis. Its activity replenishes NAD for glycolysis and can support tumor proliferation. High MDH1 can be linked to metabolic reprogramming of glioblastoma for adaptation to high ROS levels. RNH1 is a ribonuclease inhibitor that regulates RNA stability, and it has been associated with poor prognosis in glioblastoma. RNH1 antioxidant and redox homeostatic effects have also been reported in normal and malignant cells. FABP7 plays a key role in lipid metabolism, fatty acid transport, and neuronal development, and its alteration has been linked to different tumors. A reduction in FABP7 can hinder the uptake of its ligand docosahexaenoic acid (DHA) in the TME, thus limiting ROS generation [185]. These results suggested the role of these proteins as survival predictors and promising therapeutic targets for glioblastoma.

**Table 1:** Putative radiation resistance biomarkers identified by proteomics in different tumors.

Potential Biomarker	Quantitative Change	Biological Function	Tumor	Sample	Proteomic Method	Validation Method	Reference
CD166	Upregulated	Cell adhesion	NPC	Sensitive and resistant CNE2 cell secretome	iTRAQ labeling	WB	[134]
CFL2	Upregulated	Cytoskeletal remodeling					
FBN2	Downregulated	Cell adhesion, migration					
QSOX1	Downregulated	ECM remodeling, redox regulation					
MAPK15	Upregulated	DNA repair, oxidative stress response	NPC	Sensitive and resistant CNE2 cells	TMT labeling	WB	[135]
SPARC	Upregulated	ECM remodeling, EMT	NPC	Serum of responder and non-responder patients	TMT labeling	Not validated	[136]
SERPIND1	Upregulated	Coagulation, immune response					
C4B	Upregulated	Complement activation, immune evasion					
PPIB	Upregulated	Protein folding, ER stress					
FAM173A	Downregulated	Mitochondrial regulation					
LGALS7	Downregulated	Cell adhesion, apoptosis, inflammation	OSCC	FFPE tissues of responder and non-responder patients	LFQ-DDA	WB in cells and IHC in tissues	[141]
KRT17	Upregulated	Cytoskeleton organization, immune evasion	HPV+ OPSCC	FFPE tissues from patients with and without recurrence	LFQ-DIA	Not validated	[143]
MMP2	Upregulated	ECM remodeling, EMT, invasion					
S100A4	Upregulated	EMT, migration					
LMNB1	Upregulated	Chromatin remodeling, apoptosis, DNA repair					
CDS2	Downregulated	Phospholipid biosynthesis, metabolism					
GAPVD1	Downregulated	Vesicle trafficking, proliferation	HPV+ OPSCC	FFPE tissues from patients with and without recurrence	LFQ-DIA	Not validated	[143]
WDR81	Downregulated	Autophagy, stress response					
RRAS	Upregulated	Survival signaling, proliferation	OSCC	Frozen tissue of patients with and without recurrence	TMT labeling	IHC	[148]
CTSD	Upregulated	Proteolysis, apoptosis, inflammation	Triple negative breast cancer	MDA-MB-231 cells treated with single or fractionated RT	SILAC labeling	WB	[150]
GSN	Upregulated	Cytoskeleton organization, immune response					
MRC2	Upregulated	ECM remodeling, cell motility					
CHK1	Upregulated	DDR, cell cycle	ER+ breast cancer	Sensitive and resistant MCF-7 cells	Labeling and MRM	WB	[151]
CDK1	Upregulated	DDR, cell cycle					
CDK2	Upregulated	DDR, cell cycle					
TAF9	Upregulated	Transcription regulation	Triple negative and ER+ breast cancer	Sensitive and resistant MCF-7 and MDA-MB-231 cells	SILAC labeling and PRM	WB	[152]
DEK (P)	Downregulated	Chromatin remodeling, DNA repair	Triple negative breast cancer	MDA-MB-231 and MDA-MB-468 cells irradiated and treated with radiosensitizer	Phospho-proteomics	Not validated	[153]
NCL (P)	Downregulated	Ribosome biogenesis, DDR					
XRCC1 (P)	Downregulated	DNA repair					
TOP2A (P)	Downregulated	DNA topology, DDR					
PRKDC	Upregulated	DNA repair	ER+ Breast cancer	MCF-7 and T47D mammospheres treated with irradiation and doxycycline	LFQ-DDA	WB	[154]
ERK2	Upregulated	Survival signaling, proliferation	HER2+ breast cancer	Hypoxic and normoxic EVs from SKBR3 cells	SILAC labeling	Not validated	[155]
GSK3A	Upregulated	Survival signaling, apoptosis					
GSK3B	Upregulated	Survival signaling, apoptosis					
MBD4	Upregulated	DNA repair	NSCLC	Sensitive and resistant H2170 cells	TMT labeling	Not validated	[158]
TIMP3	Upregulated	ECM remodeling, invasion	NSCLC	Sensitive and resistant H2170 cells	TMT labeling	Not validated	[158]
PODXL	Upregulated	Cell adhesion, EMT					
EGFR	Upregulated	Survival signaling, proliferation, angiogenesis	NSCLC	Sensitive and resistant A549 cells	LFQ-DDA	WB	[159]
PRKCA	Downregulated	Survival signaling, proliferation					
FN1	Upregulated	ECM remodeling, migration, TME modulation	NSCLC	H460 cells with and without irradiation	TMT labeling	Not validated	[161]
THBS1	Upregulated	ECM remodeling, migration, TME modulation					
PPAT	Downregulated	Nucleotide biosynthesis, proliferation	NSCLC	Resistant H358 and H157 cells treated with radiosensitizer	SILAC labeling	WB	[162]
SERPINA1	Upregulated	Immune modulation, inflammation	NSCLC	Serum of responder and non-responder patients	LFQ-DDA	ELISA	[164]
CRP	Upregulated	Inflammation, invasion	NSCLC	Plasma of patients before and during RT	iTRAQ labeling	ELISA	[166]
LRG1	Upregulated	Angiogenesis					
ALDOA	Upregulated	Glycolysis, metabolism	Prostate cancer	Sensitive and resistant PC-3, DU145, LNCaP cells	LFQ-DDA	ALDOA validated by WB	[168]
AHSG	Upregulated	Inflammation					
VIM	Upregulated	EMT, invasion					
YWHAE	Upregulated	Stress response, cell cycle regulation					
PRDX6	Upregulated	ROS detoxification					
CD44	Upregulated	Cell adhesion, migration	Prostate cancer	Sensitive and resistant DU145 cells	LFQ-DDA	WB	[170]
ASNS	Upregulated	UPR, ER stress	Prostate cancer	22Rv1 cells with and without irradiation	LFQ-DIA	Not validated	[171]
PPAT	Downregulated	Nucleotide biosynthesis, proliferation	NSCLC	Resistant H358 and H157 cells treated with radiosensitizer	SILAC labeling	WB	[162]
SERPINA1	Upregulated	Immune modulation, inflammation	NSCLC	Serum of responder and non-responder patients	LFQ-DDA	ELISA	[164]
CRP	Upregulated	Inflammation, invasion	NSCLC	Plasma of patients before and during RT	iTRAQ labeling	ELISA	[166]
LRG1	Upregulated	Angiogenesis					
ALDOA	Upregulated	Glycolysis, metabolism	Prostate cancer	Sensitive and resistant PC-3, DU145, LNCaP cells	LFQ-DDA	ALDOA validated by WB	[168]
AHSG	Upregulated	Inflammation					
VIM	Upregulated	EMT, invasion					
YWHAE	Upregulated	Stress response, cell cycle regulation					
PRDX6	Upregulated	ROS detoxification					
CD44	Upregulated	Cell adhesion, migration	Prostate cancer	Sensitive and resistant DU145 cells	LFQ-DDA	WB	[170]
ASNS	Upregulated	UPR, ER stress	Prostate cancer	22Rv1 cells with and without irradiation	LFQ-DIA	Not validated	[171]
LDHA	Upregulated	Glycolysis, metabolism	Prostate cancer	Sensitive and resistant mouse xenograft	LFQ-DDA	WB and IHC	[172]
FTL	Upregulated	Iron homeostasis, ROS detoxification	Prostate cancer	Frozen and FFPE tissues of patients before and after RT	LFQ-DIA	WB	[173]
FGG	Upregulated	Wound healing, angiogenesis, inflammation					
ACPP	Downregulated	Signal transduction, proliferation					
TOP2A	Upregulated	DNA topology, DDR	Prostate cancer	FFPE tissues of multiresistant cancer	LFQ-DIA	Not validated	[177]
S100A8	Upregulated	Inflammation, TME modulation					
S110A9	Upregulated	Inflammation, TME modulation					
KPNA2	Upregulated	Nuclear transport, EMT					
SPON2	Upregulated	Cell adhesion, immune response					
AGR2	Downregulated	Protein folding, stress response	Prostate cancer	FFPE tissues of multiresistant cancer	LFQ-DIA	Not validated	[177]
ABCC4	Downregulated	Transport, detoxification					
ALOX15B	Downregulated	Lipid metabolism, inflammation					
S100A6	Upregulated	DNA repair, EMT	ESCC	Sensitive and resistant TE-1 and KYSE-150 cells	TMT labeling	WB	[178]
TGM2	Upregulated	Autophagy, stress response					
PYGB	Upregulated	Metabolism					
CST5	Upregulated	Migration, EMT	CRC	SW480 cells irradiated and treated with radiosensitizer	LFQ-DDA	WB in cells and IHC in xenografts	[181]
PAI1	Upregulated	Migration, EMT					
RAB5C	Upregulated	Endocytosis, DDR	Rectal cancer	Sensitive and resistant SW837 cells plus FFPE tissues of patients before and after RT	LFQ-DDA	WB in cells and IHC in tissues	[182]
XRCC5	Upregulated	DNA repair					
XRCC6	Upregulated	DNA repair					
PSME1	Upregulated	Proteostasis, survival signaling	GB	Sensitive and resistant U87MG and SF268 cells	iTRAQ labeling	WB	[184]
PSMA7	Upregulated	Proteostasis, survival signaling					
PSMB4	Upregulated	Proteostasis, survival signaling					
MDH1	Upregulated	Metabolism, redox regulation	GB	FFPE tissues and serum of short-term and long-term survivors after RT	LFQ-DIA	FABP7 validated by IHC	[185]
RNH1	Upregulated	RNA stability, ROS detoxification					
FABP7	Downregulated	Lipid metabolism, ROS detoxification					

Abbreviations: (P): phosphorylated protein; ECM (extracellular matrix); EMT (epithelial–mesenchymal transition); DDR (DNA-damage response); TME (tumor microenvironment); ROS (reactive oxygen species); UPR (unfolded protein response); ER (endoplasmic reticulum); NPC (nasopharyngeal carcinoma); OSCC (oral squamous cell carcinoma); HPV+ OPSCC (human papillomavirus-associated oropharyngeal squamous cell carcinoma); ER+ (estrogen receptor positive); HER2+ (human epithelial growth factor 2 positive); NSCLC (non-small cell lung cancer); ESCC (esophageal squamous cell carcinoma); CRC (colorectal cancer); GB (glioblastoma); FFPE (formalin-fixed, paraffin-embedded); RT (radiotherapy); EVs (extracellular vesicles); iTRAQ (isobaric tags for relative and absolute quantification); TMT (tandem mass tag); LFQ (label-free quantification); DDA (data-dependent acquisition); DIA (data-independent acquisition); SILAC (stable isotope labeling by amino acids in cell culture); MRM (multiple reaction monitoring); PRM (parallel reaction monitoring); WB (western blot); IHC (immunohistochemistry); ELISA (enzyme-linked immunosorbent assay).

## 5. Conclusions and Perspectives

Resistance to radiotherapy limits the possibility of cancer treatment through different mechanisms that confer tumor cells the capacity to enhance survival and DNA repair and evade apoptosis and immune response. Understanding the molecular features that regulate RT resistance is crucial to planning the correct treatment strategy and developing new multimodal therapeutic approaches. Clinical proteomics applied in the context of systems and precision medicine can represent a pivotal tool for the characterization of cancer patients. Specifically, it can help to stratify subjects between responders and non-responders and identify diagnostic and prognostic biomarkers and potential therapeutic targets for elucidating and overcoming radioresistance. In recent years, proteomics performed with LC-MS has been employed to study RT effects and resistance in different types of tumors, especially emphasizing the search for biomarkers. As described in this review, head and neck cancers, breast cancer, lung cancer, and prostate cancer were the most analyzed tumors, and results on colorectal cancer and glioblastoma were also added. Most of the studies were focused on comparing radiosensitive and radioresistant cells to extract deregulated proteins and pathways involved in resistance mechanisms. However, proteomic analyses of tissues and biofluids (plasma and serum) from patients treated with RT were also reported, highlighting the translatability of this approach. Different proteomic techniques were used, including label-based and label-free approaches, as well as cutting-edge methods such as DIA and PRM. Several potential biomarkers were proposed as prognosis predictors or novel therapeutic targets associated with RT resistance. Stress response, protein folding, DNA repair, inflammation, cell motility, and proliferation signaling were the most recurrent pathways modulated by the identified biomarkers, indicating their direct role in resistance and making them possible targets for novel treatments to induce radiosensitization. The validation of these proteins corroborated the proteomic results and demonstrated the capability of this approach to capture alterations in physiopathological systems with high confidence. Further characterizations are required to use these proposed biomarkers in decisive steps for clinical translation.

The identification of reliable biomarkers predictive of radioresistance has substantial implications for clinical management in radiotherapy. Integrating such biomarkers into clinical workflows would enable the stratification of patients prior to treatment, allowing for personalized therapeutic decisions and alternative or combined approaches to maximize effectiveness and outcomes.

Advancements in LC-MS technologies and software expanded the possibility of proteomics in clinical applications, although some challenges remain. Most studies were conducted on cell lines or animal models, with limited implementation for patients. Moreover, the wide range of different methods (choice of sample processing, ion source, HPLC settings, mass analyzer, acquisition method, and quantification strategy) causes an incomplete consensus and overlap in MS-based results, restricting the integration with other, more straightforward omics technologies, such as genomics and transcriptomics. Despite the increasing reliability of proteomic results, the need for validation in clinical samples and the high analysis cost hamper the application of proteomics to large-scale studies. However, rapid innovations in automated systems and data analysis are paving the way for extending proteomics in clinical practice, improving throughput, and reaching the single-cell level.

In conclusion, the results reviewed here confirm proteomics as a key component for the study of treatment response in cancer and the need to integrate this technology into personalized medicine to improve radiotherapy efficacy.
